# Impacts of Micro- and Nanoplastics on Photosynthesis Activities of Photoautotrophs: A Mini-Review

**DOI:** 10.3389/fmicb.2021.773226

**Published:** 2021-11-17

**Authors:** Yunxue Li, Xianhua Liu, Shrameeta Shinde, Jiao Wang, Pingping Zhang

**Affiliations:** ^1^School of Environmental Science and Engineering, Tianjin University, Tianjin, China; ^2^Department of Microbiology, Miami University, Oxford, OH, United States; ^3^College of Food Science and Engineering, Tianjin Agricultural University, Tianjin, China

**Keywords:** microplastics, nanoplastics, photosynthetic activity, impact, photosynthetic organisms

## Abstract

The accumulation of micro- and nanoplastics (MNPs) has attracted immense global attention due to their adverse effects on the environment. Photosynthesis, an interface between non-living matter and living organisms, is very important for both energy flow and material circulation on our planet. Increasing evidence indicates that MNPs can pose direct or indirect stress effects on photoautotrophs, however, our knowledge about them is still limited. The purposes of this mini-review are (1) to review the latest literature of the impacts of MNPs on photosynthesis activities and summarize diverse impacts of MNPs on photosynthesis activities of different photoautotrophs (green plants, microalgae, and cyanobacteria); (2) to discuss the potential action mechanisms in both aquatic and terrestrial environments; and (3) various factors contributing toward these impacts. Additionally, this review provides key future research directions for both researchers and policymakers to better understand and alleviate the environmental impacts of MNPs on our planet.

## Introduction

Microplastics (MPs) are plastics with a particle size of 5 mm or less (Van Cauwenberghe et al., 2015), and nanoplastics (NPs) are usually referred to as plastic fragments with a particle size between 1–100 nm (Ferreira et al., 2019). Both MPs and NPs can be defined as micro- and nanoplastics (MNPs), however, the definition of the particle size of these terms is still under debate (Da Costa et al., 2016; Gigault et al., 2018). At present, MNPs have been widely found in soils, rivers, and oceans around the world (Sridharan et al., 2021; Wang et al., 2021). Even in the marine environment solely, 4.85 trillion MPs are accumulated (Eriksen et al., 2014). Furthermore, the small size of NPs allows them to easily enter the bodies of predatory organisms, thereby affecting their survival activities. MNPs have been reported to act as carriers that facilitate absorption of various pollutants in the environment, altering their behaviors in both the environment (Bakir et al., 2014; Hüffer et al., 2019; Liu et al., 2021) and the organism’s body (Rochman et al., 2013). The altered degradation efficiency of pollutants and microbial diversity (Wang J. et al., 2020) can pose a potential threat to the ecosystem and human health (Wang et al., 2019).

Photosynthesis, as an interface between non-living matter and living organisms, is very important for both energy flow and material circulation on our planet. Photosynthetic organisms are an extremely important part of the ecosystem being the primary producer of organic carbon. These phototrophs convert 200 billion tons of carbon dioxide into complex organic compounds every year and generate 140 billion tons of oxygen into the atmosphere (Johnson, 2016). Therefore, the reliable operation of photosynthesis is a prerequisite for the survival of aerobic organisms at all levels. If photosynthesis is strongly disturbed, it can trigger a domino effect that will impair the stability of the ecosystem and human society.

**FIGURE 1:**
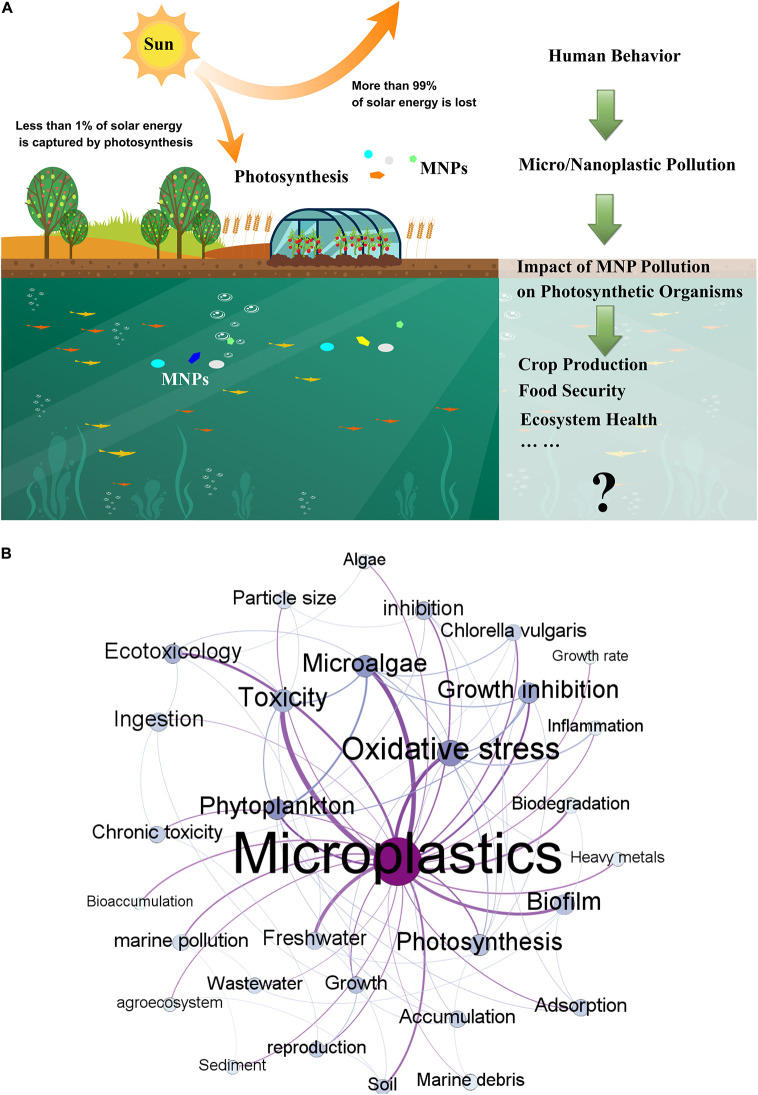
**(A)** Schematic of the importance of study of impact of MPs on photosynthetic organisms. **(B)** Co-occurrence network analysis of keywords in MPs publications. Each keyword on the map is displayed as a node, with size determined by the occurrence. Keyword relationships are shown as edges of varying thickness determined by the co-occurrence.

Photoautotrophs mainly include plants, microalgae (diatoms, phytoplankton and green algae, etc.), and cyanobacteria. The photosynthetic process involves multiple steps such as light absorption, electron transfer, photosynthetic phosphorylation, and carbon assimilation (Stanier and Cohenbazire, 1977; Johnson, 2016). Photosynthesis can be affected by environmental factors (Kaiser et al., 2015), thus when the environment is disturbed, the photosynthesis will also be affected. Diverse impacts of MNPs have been widely reported on the photosynthesis in aquatic and terrestrial photosynthetic organisms (Bhattacharya et al., 2010; Lyakurwa, 2017; Wu et al., 2019; Dong et al., 2020; Wang F. et al., 2020; Meng et al., 2021). These facts demonstrated MNPs have emerged as a potential risk for both ecosystem health and food safety (Figure 1A). However, detailed understanding of their impacts on the environment is still extremely limited.

In order to determine the research hotspots of this area, a bibliometric analysis has been conducted. Figure 1B shows the co-occurrence network analysis result of the keywords. This figure shows that the study of MNPs on phytoplankton, microalgae, growth inhibition, oxidative stress, and other toxicological aspects has attracted considerable attention. Additionally, photosynthesis, a keyword closely related to the above aspects, is one of the focuses of the MNPs research. However, the amount of work is still relatively insufficient. Current research is mainly focused on aquatic microalgae and terrestrial crops. Supplementary Tables 1, 2 list the latest research work on this topic for aquatic and terrestrial organisms, respectively.

Based on these knowledge, this mini-review serves to provide a comprehensive summary and assessment on the impacts of MNPs on photosynthetic activity, via elaborating on the following three aspects: (1) updating research progress of effect of MPS on photosynthetic activity; (2) analyzing interactions between MNPs and photoautotrophs in the aquatic and terrestrial environment; and (3) discussing the possible action mechanisms and various internal and external factors affecting the photosynthetic activity via MNPs. This is the first review on the impacts of MNPs on photosynthesis activities of phototrophs. The action modes and the roles they played in different environments were highlighted. In addition, future research directions were discussed to guide researchers and policy makers to better understand and alleviate the impacts of MNPs.

## Impact of Micro- and Nanoplastics on Photosynthesis Activity

In different environments, photoautotrophs interact with MNPs in different ways. MNPs can attach organisms and affect photosynthesis directly (Wu et al., 2019), and they can also co-load with other pollutants and change the bioavailability of other pollutants, thereby affecting photosynthesis indirectly (Zhang et al., 2018; Dong et al., 2020, 2021b). The environment in which photosynthetic organisms grow plays an important role in how they come in contact with MNPs. Therefore, this review divides photoautotrophs into aquatic and terrestrial organisms and then evaluates the impacts of MNPs on their photosynthesis, respectively. Due to the lack of studies on deep-level, such as light reaction and Calvin cycle, this review will only focus on photosynthetic pigments, photosynthetic efficiency and other intuitive parameters about photosynthetic activity.

### Impact on Photosynthetic Activity of Aquatic Photoautotrophs

At present, most work on the effects of MNPs on photosynthetic activity of aquatic photosynthetic organisms focuses on microalgae and cyanobacteria. Content of photosynthetic pigment and photosynthesis rate was normally employed as indicators to study the impact.

#### Impact on Photosynthetic Pigments

Most studies have shown that MNPs have an inhibitory effect on the content of photosynthetic pigment for aquatic photoautotrophs (Lyakurwa, 2017; Wu et al., 2019). The impact of MNPs on the photosynthetic pigment content may be affected by the species difference, MNPs concentration, particle size and type (Long et al., 2017; Zhang et al., 2017; Prata et al., 2018). For instance, Wang S. et al. (2020) found that polyvinyl chloride (PVC) inhibited the chlorophyll content in three kinds of algae, and the inhibitory effect was directly proportional to the concentration of MPs. Chen et al. (2020) demonstrated that aged polystyrene (PS) also reduced the content of chlorophyll a, chlorophyll c, and carotenoids in *Phaeodactylum tricornutum* Bohlin cells. Studies have also shown that NPs have negative effects on the photosynthetic pigments of microalgae (Bhattacharya et al., 2010; Besseling et al., 2014).

Nevertheless, there are some contradictory reports on the impact of MPs on the photosynthetic pigment content (Song et al., 2020; Su et al., 2020). The possible reason for the inconsistent phenomena may be that the photoautotrophs can slowly acclimatize themselves to the prolonged MNPs stress and upregulate the synthesis of photosynthetic pigments. In addition, the different operations and procedures used by different authors may also be the reasons for the above phenomena.

#### Impact on Photosynthetic Efficiency

Various studies demonstrated that MNPs exhibit an inhibitory effect on the photosynthetic efficiency of microalgae (Bhattacharya et al., 2010; Zhang et al., 2017; Wu et al., 2019; Zhao et al., 2019; Wang C. et al., 2020; Dong et al., 2021a). In some cases, the inhibitory effect on photosynthetic efficiency was debilitated if the concentration of plastic particles was maintained and the photosynthetic microorganisms were given time to acclimate. For example, Mao et al. (2018) showed that photosynthesis of *Chlorella pyrenoidosa* was inhibited during the first 6–8 days after exposure to nano-scale PS but recovered after that. The maximal photochemical efficiency of photosystem (Fv/Fm) of *Chlamydomonas reinhardtii* was reduced after exposure to nano-scale PS, but the inhibition effect began to diminish on the tenth day (Li S. et al., 2020). Chen et al. (2020) found that aged micron PS can also make the maximum photochemical efficiency, photosynthetic activity and photoprotective ability of *Phaeodactylum tricornutum* Bohlin behaved adaptation to stress.

There are also some studies found that exposure to some types of MNPs does not have a significant impact on the photosynthesis of aquatic photoautotrophs, and even can promote photosynthesis (Chae et al., 2019). For example, amino-modified polystyrene (PS-NH_2_) are reported have no significant effect on the photosynthesis of *Chaetoceros eogracile* (Seoane et al., 2019), and the effect of 1 μm PS on the Fv/Fm of Microcystis aeruginosa can be neglected (Wu et al., 2021). Sjollema et al. (2016) reported neither PS nor negatively charged carboxylated polystyrene has a significant effect on the photosynthesis of *Dunaliella tertiolecta*. These studies demonstrated that the impacts of MPs on the photosynthetic efficiency can also be affected by the species difference, MPs concentration, particle size and type, and their impacts need to be analyzed individually.

### Impact on Photosynthetic Activity of Terrestrial Photoautotrophs

At present, studies on the influence of MNPs on the growth of terrestrial plants mostly focus on grain crops such as corn and wheat, as well as cash crops such as lettuce and cucumber. Current research focuses on roots that are in direct contact with soil and MNPs as a part of the maximal impact from MPs accumulation. A study has pointed that sub-micrometer and micrometer-sized plastics can penetrate plant root and be transported from the roots to the shoots (Li L. et al., 2020). However, the exact conclusions about whether all NPs can enter and be transported in plants need to be further studied.

#### Impact on Photosynthetic Pigment and Other Parameters

Many studies showed that MNPs had inhibitory effects on the pigment content in the terrestrial photoautotrophs. Wang F. et al. (2020) showed that MPs formed by polylactic acid (PLA), which accounts for 0.1, 1, and 10% of the soil, respectively, can reduce the chlorophyll content in maize leaves. The relative chlorophyll content of Common bean (*Phaseolus vulgaris* L.) leaves was significantly reduced after being exposed to low density polyethylene (LDPE) (Meng et al., 2021). On contrary, other studies have reported increased pigment content in the leaves of terrestrial photoautotrophs under the exposure of MNPs. For example, in the study of Liao et al. (2019), after wheat (*Triticum aestivum*) was exposed to PS, the photosynthetic pigment content increased slightly. Ren et al. (2021) studied the effect of PS on the photosynthesis of Flowering Chinese cabbage (*Brassica rapa* syn. *Campestris* L. ssp. *Chinensis* var. *utilis* Tsen et Lee) in soil. Their research showed that after adding PS to the soil, the content of chlorophyll a in flowering Chinese cabbage increased, and the content negatively correlated with the particle size of PS. There are also studies showing that adding MNPs has no significant effect on the chlorophyll content (Bosker et al., 2019).

#### Combination Impacts of Micro- and Nanoplastics and Other Pollutants

Taking into account the interaction between MNPs and other substances, studies are showing that MNPs can affect the toxicity or bioavailability of other pollutants. A recent study investigated the combined effect of MPs and dibutyl phthalate (DBP) on the photosynthesis of hydroponic red lettuce (*Lactuca sativa* L. Red Sails) (Dong et al., 2021b). Their study compared the bioavailability of DBP before and after the addition of PS and showed that PS can affect the bioavailability of DBP. Another study found that adding a low concentration of MPs (0.04 g/L PS, 0.1 g/L polytetrafluoroethylene (PTFE), respectively) with As(III) can reduce the negative effects of As(III) on Rice (*Oryza sativa*), but the impact of As(III) became higher than the application of As(III) alone under the adding of 0.2 g/L PS or PTFE, respectively (Dong et al., 2020). These studies demonstrated that the role played by MNPs in the interaction of photosynthetic organisms with other substances is not static, and may often show different effects with changes in concentration or particle size.

### Possible Acting Mechanisms and Influencing Factors

**FIGURE 2:**
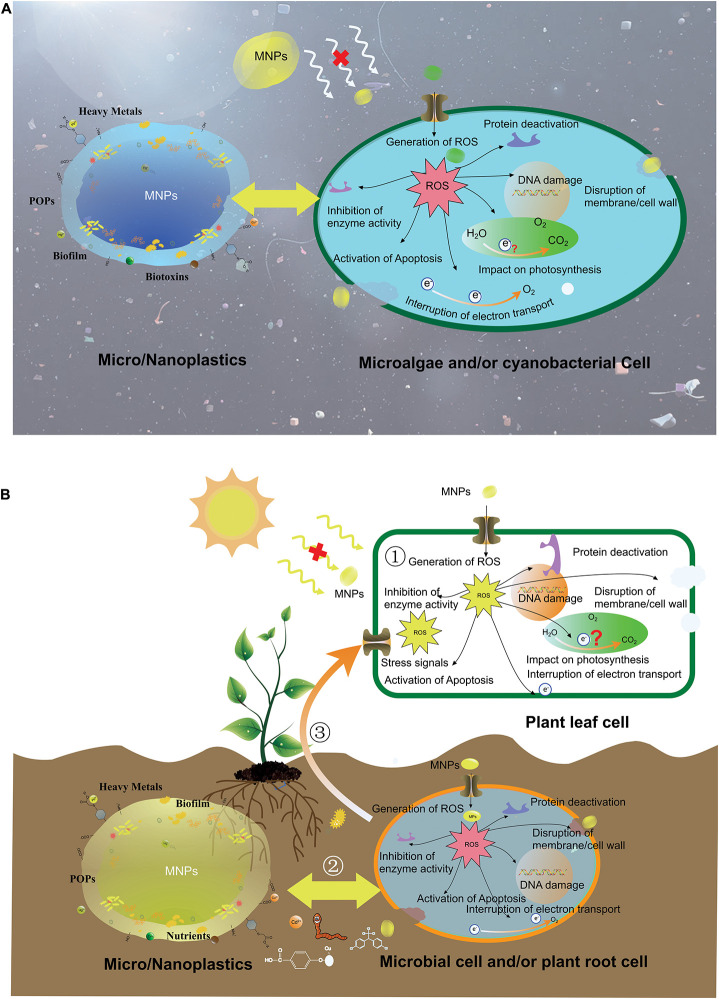
Schematics of the potential impacts of MPs on aquatic photosynthetic organisms **(A)** and terrestrial photosynthetic organisms **(B)**.

MNPs can directly/indirectly contact photoautotrophs, and the schematics of their interactions are shown in Figure 2.

In aquatic environment, MNPs can attach to the surface of microalgae or other aquatic phototrophs, influencing the access to light energy and possibly causing physical damage to cells (Figure 2A). Some small particles can enter the cell, resulting in the generation of ROS, leading to a series of oxidative stress reactions. These ROS may damage organelles such as chloroplasts thereby affecting photosynthesis directly. Due to the diversity and complexity of the factors affecting the growth of organisms, the specific mechanisms of the influence of MNPs on algae are still difficult to be accurately judged. The difference in the gene expression of microalgae, the type, concentration, shape of MNPs, and the length of cultivation time may all result in different responses to MNPs. Thus, most of the current conclusions are speculated based on experiments. For example, according to a recent study, the differences in microalgae gene expression may cause different responses to the same MNPs (Song et al., 2020). Wu et al. (2019) reported the specific inhibition effects on photosynthesis were different when the same microalgae were exposed to different MNPs, respectively, although the general trend was approximately the same. And according to the study of Gao et al. (2021), it can be speculated that different shapes of MNPs have a different impact on microalgae. They believe that the MNPs particles with irregular shape seems to be easier to damage the microalgae than the bead due to its irregular shape. These speculations are reasonable, but in the future, a large number of repeated experiments are still needed to obtain systematic conclusions on the interaction mechanism of MNPs and microalgae. In the aquatic environment, the combination of MNPs with heavy metals, persistent organic pollutants (POPs), and other pollutants is also a common phenomenon. If considering the co-loading of them, the mechanism impact on photosynthesis of aquatic photosynthetic organisms will be more complicated.

In terrestrial environment, although MNPs cannot directly interact with leaf cells, they can affect the photosynthesis of terrestrial photosynthetic organisms in other ways. Figure 2B shows the interactions of MNPs and terrestrial photosynthetic organisms. When MNPs in the soil come into contact with plant roots, they may affect the morphology and structural integrity of root cells, the absorption, transport of nutrients, and even affect gene expression. Plants receiving external stimulus signals may produce a stress response, which affects the progress of normal cellular activities such as the extent of photosynthesis. Therefore, the content of photosynthetic pigments, intercellular carbon dioxide concentration, stomatal conductance, and other parameters will be influenced accordingly. Under this circumstance, plants may self-regulate, such as increasing the leaf area, to diminish the negative impact of the reduction of chlorophyll content. A recent study speculated that under different concentrations of MNPs, the roots of plants have different adsorption effects on them (An et al., 2021). MNPs tend to agglomerate when the concentration is high so that their adsorption by roots will be lowered thereby diminishing the photosynthetic stress. If MNPs enter the plant body through root cells, they will have a more direct impact on plant growth and photosynthesis. However, there is still controversy about whether MNPs can enter and get transported inside plants. Furthermore, there are many kinds of pollutants, such as heavy metals, POPs, nutrients, and microbes in the soil. All these substances may combine with MNPs, and this combination can change their toxicity or bioavailability.

## Conclusion and Prospects

MNPs have spread all over the world. Due to the lack of scientific and efficient degradation methods, it will gradually be enriched in the environment. The importance of photoautotrophs to ecosystems and human society is unquestionable, and their photosynthesis is a key part of material circulation, energy flow, and sustenance of life. Therefore, it is necessary to explore the influence of MNPs on photosynthetic organisms. Current research has demonstrated that exposure of phototrophs to MNPs can lead to altered photosynthetic activity. However, it is still difficult to quantify the impact and determine their acting mechanisms.

In the future, research can be continued from the following aspects:

(1) The factors affecting the impacts should be systematically explored at different levels and the working mechanism need to be clarified by using a combination of various approaches. For example, time-lapse study of impacts of MNPs on the population dynamics and regulation.
(2) New methods and instrumentation should be developed to overcome the challenges of instrumental limitations and complexity of the environmental system. For example, the detection and toxicity evaluation of NPs in soils and natural water bodies.
(3) The fluctuations in the microbial community at the site of MNPs accumulation in the absence or presence of MPs need to be further studied. This will help to identify if there are other interactive partners of phototrophs in the ecosystem that allow them to thrive this stress.
(4) To facilitate comparability of data between studies and expand the lab-scale findings to real-word situations, more field studies need to be conducted and operation standards for MNP sampling and analysis are urgently needed.
(5) Effective policies, technical measures and monitoring systems are also required to reduce the risks of MNPs and secure food safety. Ways to accelerate the removal of MNPs are needed to reduce the accumulation of MNPs in the environment.

## Author Contributions

YL: investigation, data curation, and writing—original draft. XL: conceptualization, supervision and writing—review and editing. SS: conceptualization and writing—review and editing. JW: resources, investigation, and data curation. PZ: resources, investigation, and data curation. All authors contributed to the article and approved the submitted version.

## Conflict of Interest

The authors declare that the research was conducted in the absence of any commercial or financial relationships that could be construed as a potential conflict of interest.

## Publisher’s Note

All claims expressed in this article are solely those of the authors and do not necessarily represent those of their affiliated organizations, or those of the publisher, the editors and the reviewers. Any product that may be evaluated in this article, or claim that may be made by its manufacturer, is not guaranteed or endorsed by the publisher.
