# Supplementary material for: Impacts of Micro- and Nanoplastics on Photosynthesis Activities of Photoautotrophs: A Mini-Review
Source: Front Microbiol. 2021 Nov 17;12:773226. doi: 10.3389/fmicb.2021.773226 (PMC8660080; doi:10.3389/fmicb.2021.773226)
Supplement: Supplementary file 1 [file Data_Sheet_1.docx]

Supplementary Material

Impacts of micro- and nanoplastics on photosynthesis activities of photoautotrophs: A mini-review

Yunxue Li^1^, Xianhua Liu^1*^, Shrameeta Shinde^2^, Jiao Wang^1^, Pingping Zhang^3^

^1^School of Environmental Science and Engineering, Tianjin University, Tianjin, 300354, PR China

^2^Department of Microbiology, Miami University, Oxford, OH, 45056, USA

^3^College of Food Science and Engineering, Tianjin Agricultural University, Tianjin, 300384, PR China

*** Correspondence:**Xianhua Liu
[lxh@tju.edu.cn](mailto:lxh@tju.edu.cn)

**Table Captions**

**Table S1** Effects of MNPs on photosynthesis in aquatic organisms.

**Table S2** Effects of MNPs on photosynthesis in terrestrial organisms.

# Tables

**Table S1** Effects of MNPs on photosynthesis of aquatic organisms.

| MNPs types | Size/μm | Concentration/ (mg/L) | Types of aquatic photosynthetic organisms | Effect Criteria | Result | Reference |
| --- | --- | --- | --- | --- | --- | --- |
| PP | 10-90 | 5-500 | *Chlorella (C.) pyrenoidosa* | Chlorophyll a | Inhibition after 3 days | (Wu et al., 2019) |
|  |  |  |  | Photosynthetic activity | Decreased significantly at first and then increased(c>5 mg/L) |  |
| PP |  |  | *Microcystis (M.) flosaquae* | Chlorophyll a | Inhibition after 2 or 3 days |  |
|  |  |  |  | Photosynthetic activity | Slightly lower in the 500 mg/L group, but no significant difference in the other groups. |  |
| PVC | 10-90 | 5-500 | *Chlorella (C.) pyrenoidosa* | Chlorophyll a | Inhibition after 5 days |  |
|  |  |  |  | Photosynthetic activity | Decreased significantly at first and then increased |  |
| PVC |  |  | *Microcystis (M.) flosaquae* | Chlorophyll a | Inhibition after 2 or 3 days |  |
|  |  |  |  | Photosynthetic activity | Significant inhibition in the 500 mg/L group, but no significant difference in the other groups. |  |
| PS（+, -） | 0.02 | 0.08-0.58 | *Chlorella sp.* | Photosynthesis | Significantly reduced when the concentration>1.8 mg/L | (Bhattacharya et al., 2010) |
|  |  |  |  | ROS production | Higher, especially in (+) |  |
|  |  |  | *Scenedesmus sp.* | Photosynthesis | Decrease up to 40% |  |
|  |  |  |  | ROS production | Higher, especially in (+) |  |
| PS | 0.07 | 44-1100 | *Scenedesmus obliquus* | Chlorophyll content | Decrease>100 mg/L | (Besseling et al., 2014) |
|  |  |  |  | Photosynthesis genes | No effect |  |
| PS | 2 | 0.004 | *Tisochrysis lutea* | Chlorophyll content | No effect | (Long et al., 2017) |
| PS | 10 | 75–7500 | *Rhodomonasbaltica* | Chlorophyll content | Significant decrease | (Lyakurwa, 2017) |
| PE | 1-5 | 0.75-48 | *Tetraselmis chuii* | Chlorophyll content | Decreased at 0.9 and 2.1 mg/ L | (Prata et al., 2018) |
| PS | 0.3-0.6 | 5, 25, 50, 100 | *Chlamydomonas reinhardtii* | Chlorophyll a fluorescence | Decreased and recovered after 10 days | (Li et al., 2020b) |
|  |  |  |  | Fv/Fm | Inhibition, but weaken after the tenth day |  |
| PS | 1 | 5 | *（endosymbiotic dinoflagellate）Cladocopium goreaui* | Chlorophyll a content | Increase and more significant at 7th day | (Su et al., 2020) |
|  |  |  |  | Fv/Fm | No significant effect |  |
| PS | 1 | 5 | *Microcystis aeruginosa* | Fv/Fm | Inhibition before 72h, promote higher at 96h | (Wu et al., 2021) |
|  | 0.1 |  |  |  | No significant effect |  |
| PVC | 1 | 0, 25, 50, 100, 200 | *Phaeodactylum tricornutum (B255)* | Chlorophyll content | Inhibition | (Wang et al., 2020d) |
|  |  |  | *Chaetoceros gracilis (B13)* | Chlorophyll content | Inhibition |  |
|  |  |  | *Thalassiosira sp. (B280)* | Chlorophyll content | Inhibition |  |
|  |  |  |  | Fv/Fm | Decrease |  |
| PS（Aging） |  | 0, 10, 20, 50, 100 | *Phaeodactylum tricornutum Bohlin* | Chlorophyll a | Decrease | (Chen et al., 2020) |
|  |  |  |  | Chlorophyll c | Decrease |  |
|  |  |  |  | Carotenoid content | Decrease |  |
|  |  |  |  | Fv/Fm | Adaptation after stress |  |
|  |  |  |  | Light protection ability | Adaptation after stress |  |
|  |  |  |  | Photosynthetic activity | Adaptation after stress |  |
| PS | 0.05, 0.5, 6 | 25, 250 | *Dunaliella tertiolecta* | Photosynthesis | No effect | (Sjollema et al., 2016) |
| PS（-） | 0.5 | 25, 250 | *Dunaliella tertiolecta* | Photosynthesis | No effect |  |
| PVC | 1 | 5, 50 | *Skeletonema costatum* | Chlorophyll content | Decrease | (Zhang et al., 2017) |
|  |  |  |  | Photosynthetic efficiency | Decrease |  |
| PE | 180-212 | 50, 100, 150,  200; 250, 300, 350 | *Dunaliella salina* | Photosynthesis | Significant increase | (Chae et al., 2019) |
| PS-NH_2_ | 2 | 2.5 | *Chaetoceros eogracile* | Photosynthesis | No effect | (Seoane et al., 2019) |
| PVC | 1 | 5, 25, 50, 100 | *Karenia mikimotoi* | Photosynthesis | Significant inhibition | (Zhao et al., 2019) |
| PE, PP, PET, PVC |  |  | *Chlorella* | Chlorophyll content | Increased first and then decreased | (Song et al., 2020) |
|  |  |  |  | Carotenoid content | Decreased first but then slowly rising (still lower than the control) |  |
|  |  |  |  |  |  |  |
|  |  |  |  |  |  |  |
| PE, PET |  |  | *Phaeodactylum tricornutum* MASCC-0025 | Chlorophyll concentration | Rising, more siginificant after 48h |  |
| PP, PVC |  |  |  |  | Decrease |  |
| PS+As | 5 μm,10 nm |  | *Chlamydomonas  reinhardtii* | Photosynthetic rate | Inhibition | (Dong et al., 2021a) |
|  |  |  |  | Chlorophyll content | Decreased significantly in 3nd-7th day |  |
| PS | 0.1 | 10, 50, 100 | *Chlorella pyrenoidosa* | Fv/Fm | Maximum inhibition rate on day8, but weakened after that | (Mao et al., 2018) |
|  | 1 |  |  |  | Maximum inhibition rate on day6, but weakened after that |  |
| PE, PA, PS |  |  | *Chlorella pyrenoidosa* | Fv/Fm | Inhibition | (Yang et al., 2020) |

**Table S2** Effects of MNPs on photosynthesis of terrestrial organisms.

| MNPs types | Size | Concentration | Types of terrestrial photosynthetic organisms | Effect Criteria | Result | Reference |
| --- | --- | --- | --- | --- | --- | --- |
| PLA |  | 0.1%, 1%, 10% | Maize (*Zea mays L.*) | Chlorophyll content | Decrease | (Wang et al., 2020b) |
| PS,PTFE+As(Ⅲ) |  |  | Rice (*Oryza sativa*) | Photosynthesis | Inhitition | (Dong et al., 2020) |
| LDPE-MPs | 724 ± 56.0 cm^2^ | 1.00% | Common bean (*Phaseolus vulgaris L.*) | Leaf area | Significantly higher | (Meng et al., 2021) |
|  | 27.2 ± 2.34 cm^2^ | 0.50% |  | Leaf relative chlorophyll content | Significantly lower |  |
| Bio-MPs |  | 2.00% |  | Leaf area | Significantly lower |  |
|  |  | 2.50% |  | Leaf relative chlorophyll content | Significantly higher |  |
| PVC | <18 μm |  | Cucumber seedlings | Carotenoids | Significantly improved the effect of Cd | (Li et al., 2020c) |
|  | 18-150 μm |  |  | Light absorption | Enhanced |  |
| PVC | <15 μm | 1.62 g/kg、2.70 g/kg | Soybean seedlings | Leaf area | Inhibition first and then recovery | (An et al., 2021) |
| PS+DBP | 0.1-0.4 μm，  10-15 μm |  | Green leaf lettuce (*Lactuca sativa L.*) | Net photosynthetic rate | Decreased | (Wang et al., 2020a) |
|  |  |  |  | Stomatal conductance | Decreased |  |
|  |  |  |  | Transpiration rate | Decreased |  |
|  |  |  |  | Intracellular CO_2_  concentration | Increased |  |
|  |  |  |  | Chlorophyll content | Decreased significantly |  |
| PS | 5, 10 μm | 1% | Wheat (*Triticum aestivum*) | Photosynthetic pigment | Increased slightly | (Liao et al., 2019) |
| HDPE | 102.6 μm | 0.10% | *Lolium perenne* | Chlorophyll a/Chlorophyll b | Increased | (Boots et al., 2019) |
| PLA | 65.6 μm | 0.10% |  |  | Increased |  |
| Green fluorescent plastic particles (Fluoro-Max Green Fluorescent Polymer Microspheres) | 50, 500, 4800 nm |  | *Lepidium sativum* | Chlorophyll a, chlorophyll b | No significant impact | (Bosker et al., 2019) |
| PS | 5 μm, 70 nm | 10 mg/kg | Flowering Chinese cabbage (*Brassica Nrapa* syn. *Campestris* L. ssp. *Chinensis* var. *utilis* Tsen et Lee) | Chlorophyll | Increased | (Ren et al., 2021) |
|  |  |  |  | Net photosynthetic rate | No effect |  |
| PS+DBP |  |  | Red lettuce (*Lactuca sativa* L. Red Sails) | Photosynthetic rate | Decreased significantly | (Dong et al., 2021b) |
|  |  |  |  | Stomatal conductance |  |  |
|  |  |  |  | Transient transpiration rate |  |  |
|  |  |  |  | Intercellular CO_2_  concentration | Increased |  |

DBP: dibutyl phthalate, HDPE: High Density Polyethylene, LDPE: Polyethylene, PA: Polyamide, PE: Polyethylene, PET: Polyethylene Terephthalate, PLA: Polylactic Acid, PP: Polypropylene, PS: Polystyrene, PVC: Polyvinyl Chloride, PS(+): Positively charged carboxylated polystyrene, PS(-): Negatively charged carboxylated polystyrene, PS-NH_2_: Amino-modified Polystyrene, PTFE: Polytetrafluoroethylene

**References**

An, J., Liu, H., Zheng, Y., Cheng, J., and Song, C. (2021). Effects of soil microplastics residue on soybean seedlings growth and the physiological and biochemical characteristics. Journal of Sichuan Agricultural University*.* 39(01), 41-46+113. doi: 10. 16036/j. issn. 1000-2650. 2021. 01. 007

Besseling, E., Wang, B., Lürling, M., and Koelmans, A. A. (2014). Nanoplastic Affects Growth of S. obliquus and Reproduction of D. magna. Environ. Sci. Technol. 48(20), 12336-12343. doi: 10.1021/es503001d

Bhattacharya, P., Lin, S., Turner, J. P., and Ke, P. C. (2010). Physical Adsorption of Charged Plastic Nanoparticles Affects Algal Photosynthesis. The Journal of Physical Chemistry C*.* 114(39), 16556-16561. doi: 10.1021/jp1054759

Boots, B., Russell, C. W., and Green, D. S. (2019). Effects of Microplastics in Soil Ecosystems: Above and Below Ground. Environ. Sci. Technol. 53(19), 11496-11506. doi: 10.1021/acs.est.9b03304

Bosker, T., Bouwman, L. J., Brun, N. R., Behrens, P., and Vijver, M. G. (2019). Microplastics accumulate on pores in seed capsule and delay germination and root growth of the terrestrial vascular plant Lepidium sativum. Chemosphere*.* 226, 774-781. doi: 10.1016/j.chemosphere.2019.03.163

Chae, Y., Kim, D., and An, Y. (2019). Effects of micro-sized polyethylene spheres on the marine microalga Dunaliella salina: Focusing on the algal cell to plastic particle size ratio. Aquat. Toxicol. 216, 105296. doi: 10.1016/j.aquatox.2019.105296

Chen, Z., Hong, Y., Hao, L., and Li, L. (2020). Effects of aging microplastics on the growth and photosynthetic physiology of Phaeodactylum tricornutum bohlin. Environ. Sci. Technol. 43(03), 30-37. doi: 10.19672/j.cnki.1003-6504.2020.03.004

Dong, Y., Gao, M., Qiu, W., and Song, Z. (2021a). Effects of microplastic on arsenic accumulation in Chlamydomonas reinhardtii in a freshwater environment. J. Hazard. Mater. 405, 124232. doi: 10.1016/j.jhazmat.2020.124232

Dong, Y., Gao, M., Song, Z., and Qiu, W. (2020). Microplastic particles increase arsenic toxicity to rice seedlings. Environ. Pollut. 259, 113892. doi: 10.1016/j.envpol.2019.113892

Dong, Y., Song, Z., Liu, Y., and Gao, M. (2021b). Polystyrene particles combined with di-butyl phthalate cause significant decrease in photosynthesis and red lettuce quality. Environ. Pollut. 278, 116871. doi: 10.1016/j.envpol.2021.116871

Li, S., Wang, P., Zhang, C., Zhou, X., Yin, Z., and Hu, T., et al. (2020b). Influence of polystyrene microplastics on the growth, photosynthetic efficiency and aggregation of freshwater microalgae Chlamydomonas reinhardtii. Sci. Total Environ. 714, 136767. doi: 10.1016/j.scitotenv.2020.136767

Li, Z., Li, Q., Li, R., Zhao, Y., Geng, J., and Sun, Y., et al. (2020c). Physiological response of cucumber seedlings to microplastics and cadmium. Journal of Agro-Environment Science*.* 39(05), 973-981. doi: 10.11654/jaes.2019-1241

Liao, Y., Nazygul·jahitbek, Li, M., Wang, X., and Jiang, L. (2019). Effects of Microplastics on the Growth， Physiology， and Biochemical Characteristics of Wheat (Triticum aestivum). Environmental Science*.* 40(10), 4661-4667. doi: 10.13227 /j.hjkx.201903113

Long, M., Paul-Pont, I., Hégaret, H., Moriceau, B., Lambert, C., and Huvet, A., et al. (2017). Interactions between polystyrene microplastics and marine phytoplankton lead to species-specific hetero-aggregation. Environ. Pollut. 228, 454-463. doi: 10.1016/j.envpol.2017.05.047

Lyakurwa, D. J. (2017). Uptake and effects of microplastic particles in selected marine microalgae species; Oxyrrhis marina and Rhodomonas baltica. Norwegian University of Science and Technology. pp 1-65.

Mao, Y., Ai, H., Chen, Y., Zhang, Z., Zeng, P., and Kang, L., et al. (2018). Phytoplankton response to polystyrene microplastics: Perspective from an entire growth period. Chemosphere*.* 208, 59-68. doi: 10.1016/j.chemosphere.2018.05.170

Meng, F., Yang, X., Riksen, M., Xu, M., and Geissen, V. (2021). Response of common bean (Phaseolus vulgaris L.) growth to soil contaminated with microplastics. Sci. Total Environ. 755, 142516. doi: 10.1016/j.scitotenv.2020.142516

Prata, J. C., Lavorante, B. R. B. O., B. S. M. Montenegro, M. D. C., and Guilhermino, L. (2018). Influence of microplastics on the toxicity of the pharmaceuticals procainamide and doxycycline on the marine microalgae Tetraselmis chuii. Aquat. Toxicol. 197, 143-152. doi: 10.1016/j.aquatox.2018.02.015

Ren, X., Tang, J., Wang, L., and Liu, Q. (2021). Microplastics in soil-plant system: effects of nano/microplastics on plant photosynthesis, rhizosphere microbes and soil properties in soil with different residues. Plant Soil*.* 462(1-2), 561-576. doi: 10.1007/s11104-021-04869-1

Seoane, M., González-Fernández, C., Soudant, P., Huvet, A., Esperanza, M., and Cid, Á., et al. (2019). Polystyrene microbeads modulate the energy metabolism of the marine diatom Chaetoceros neogracile. Environ. Pollut. 251, 363-371. doi: 10.1016/j.envpol.2019.04.142

Sjollema, S. B., Redondo-Hasselerharm, P., Leslie, H. A., Kraak, M. H. S., and Vethaak, A. D. (2016). Do plastic particles affect microalgal photosynthesis and growth? Aquat. Toxicol. 170, 259-261. doi: 10.1016/j.aquatox.2015.12.002

Song, C., Liu, Z., Wang, C., Li, S., and Kitamura, Y. (2020). Different interaction performance between microplastics and microalgae: The bio-elimination potential of Chlorella sp. L38 and Phaeodactylum tricornutum MASCC-0025. Sci. Total Environ. 723, 138146. doi: 10.1016/j.scitotenv.2020.138146

Su, Y., Zhang, K., Zhou, Z., Wang, J., Yang, X., and Tang, J., et al. (2020). Microplastic exposure represses the growth of endosymbiotic dinoflagellate Cladocopium goreaui in culture through affecting its apoptosis and metabolism. Chemosphere*.* 244, 125485. doi: 10.1016/j.chemosphere.2019.125485

Wang, C., Liu, Y., Song, Z., and Gao, M. (2020a). Effects of microplastics and DBP on photosynthesis and nutritional quality of lettuce. Journal of Agro-Environment Science*.* 03(40), 518-516. doi: 10.11654/jaes.2020-1134

Wang, F., Zhang, X., Zhang, S., Zhang, S., and Sun, Y. (2020b). Interactions of microplastics and cadmium on plant growth and arbuscular mycorrhizal fungal communities in an agricultural soil. Chemosphere*.* 254, 126791. doi: 10.1016/j.chemosphere.2020.126791

Wang, S., Wang, Y., Liang, Y., Cao, W., Sun, C., and Ju, P., et al. (2020d). The interactions between microplastic polyvinyl chloride and marine diatoms: Physiological, morphological, and growth effects. Ecotox. Environ. Safe. 203, 111000. doi: 10.1016/j.ecoenv.2020.111000

Wu, D., Wang, T., Wang, J., Jiang, L., Yin, Y., and Guo, H. (2021). Size-dependent toxic effects of polystyrene microplastic exposure on Microcystis aeruginosa growth and microcystin production. Sci. Total Environ. 761, 143265. doi: 10.1016/j.scitotenv.2020.143265

Wu, Y., Guo, P., Zhang, X., Zhang, Y., Xie, S., and Deng, J. (2019). Effect of microplastics exposure on the photosynthesis system of freshwater algae. J. Hazard. Mater. 374, 219-227. doi: 10.1016/j.jhazmat.2019.04.039

Yang, W., Gao, X., Wu, Y., Wan, L., Tan, L., and Yuan, S., et al. (2020). The combined toxicity influence of microplastics and nonylphenol on microalgae Chlorella pyrenoidosa. Ecotox. Environ. Safe. 195, 110484. doi: 10.1016/j.ecoenv.2020.110484

Zhang, C., Chen, X., Wang, J., and Tan, L. (2017). Toxic effects of microplastic on marine microalgae Skeletonema costatum: Interactions between microplastic and algae. Environ. Pollut. 220, 1282-1288. doi: 10.1016/j.envpol.2016.11.005

Zhao, T., Tan, L., Huang, W., and Wang, J. (2019). The interactions between micro polyvinyl chloride (mPVC) and marine dinoflagellate Karenia mikimotoi: The inhibition of growth, chlorophyll and photosynthetic efficiency. Environ. Pollut. 247, 883-889. doi: 10.1016/j.envpol.2019.01.114
